# Male Presence can Increase Body Mass and Induce a Stress-Response in Female Mice Independent of Costs of Offspring Production

**DOI:** 10.1038/srep23538

**Published:** 2016-03-23

**Authors:** Michael Garratt, Anthony J. Kee, Rupert Palme, Robert C. Brooks

**Affiliations:** 1 Evolution and Ecology Research Group and School of Biological, Earth and Environmental Sciences, The University of New South Wales, Sydney, NSW 2052, Australia; 2 Department of Pathology, University of Michigan Medical School, Ann Arbor, MI 48109, United States; 3 Neuromuscular and Regenerative Medicine Unit, School of Medical Sciences, The University of New South Wales, Sydney, NSW 2052, Australia; 4 Department of Biomedical Sciences/Unit of Physiology, Pathophysiology and Experimental Endocrinology, University of Veterinary Medicine Vienna, A-1210 Vienna, Austria

## Abstract

Sexual reproduction in animals requires close interactions with the opposite sex. These interactions may generate costs of reproduction, because mates can induce detrimental physiological or physical effects on one another, due to their interest in maximising their own fitness. To understand how a male’s presence influences aspects of female physiology implicated in reproductive costs in mice, independent of offspring production, we paired females with vasectomised, castrated or intact males, or other females. Being paired with a male, irrespective of his gonadal status, increased female weight. This effect was transient in females paired with castrated males but more persistent in those with vasectomised males. Those paired with males also showed an increase in corticosterone, suggesting an increased stress response. However, this was dependent on the gonadal status of the male housing partner, since those housed with vasectomised males had lower corticosterone than those with castrated males. Altered energy metabolism was only detectable in pregnant females, and oxidative stress was not consistently affected by a female’s housing partner. These results suggest that a male’s presence alters female weight, and stresses associated with reproduction could be induced by simply the presence of a male, but reduced by mating and/or being solicited to mate.

Sexual reproduction in animals, by its nature, requires interactions with members of the opposite sex; at the very least, close proximity interactions for the mixing of gametes, but often sustained close contact during mating, post-copulatory guarding, and any subsequent joint parental care. As a consequence, interactions with members of the opposite sex can be rewarding. Allowing rodents to mate in a particular area can induce a conditioned place preference. Mating can also have physiological benefits, which in *Drosophila melanogaster* males have even been shown to increase lifespan, when compared to individuals that can sense opposite sex individuals but not mate^1^.

In spite of the necessity of mating, the interests of both parties in many of these interactions are unequal^2^. Reproduction is a costly process and sexual conflict is expected over whether and how often to mate, how many offspring to have, and over how much to invest in those offspring^3^. Males in many species strive to overcome female reluctance to mate by harassing and coercing females into mating^4^,^5^. These interactions can elevate costs of reproduction for females, and increase the stress associated with fertilisation and offspring production^3^. In insects, these costs of male interactions are obvious in the substantial damage that some males induce during traumatic inseminations^6^,^7^. In mammals, males are also expected to increase the costs of reproduction to females, through their mating behaviour, their threat of infanticide, and their genes, over and above that which females suffer from normal parental care^8^. It’s also notable that while mating is rewarding for female rodents, reward responses only occur in females when they can control the pace of mating and have a refuge from male harassment to mate^9^. In spite of these insights, understanding which costs of reproduction are generated by male interactions and which are generated by offspring production are often complicated by the fact that interactions with males lead to fertilisation, and costs of male presence are usually tied to the costs of paternal care.

To distinguish the physiological effects of male presence and of mating from the costs of pregnancy and reproduction in mice, we experimentally paired females each with one of four kinds of companion individual: another female, a castrated male, a vasectomized male, or an intact male who had undergone sham vasectomy surgery. We left focal females with their assigned companion for a three to four week period, until just before females housed with intact males gave birth to young, at which point all housing partners were removed. Within two weeks of castration male mice stop showing most sexual behaviours^10^, including mounts, intromissions and ejaculation^10^,^11^,^12^, whereas vasectomized males continue to show normal levels of sexual motivation and mating, and sham vasectomized males additionally can impregnate females. The effect of mating, including the impacts of seminal fluid transfer, can therefore be assessed, independent of the costs of reproduction, by comparing females paired to the castrated and vasectomized males. The costs of reproduction can be estimated by comparing females paired to vasectomized and intact males. And the costs of male presence can be estimated by comparing females paired to castrated males with those paired with a female companion.

Various physiological changes occur with reproduction and may be associated with either rewards or costs of mating. Glucocorticoids can help adapt metabolism to the requirements of reproductive allocation, with levels increasing during pregnancy in mice^13^,^14^, but their production in some contexts can be harmful, and has been highlighted as a potential physiological cost of male harassment and sexual cohesion^15^. Such effects have been noted in pregnant and lactating chacma baboons (*Papio hamadryas ursinus*) when immigrant males take over a natal area^16^. Increased energy expenditure represents one of the most widespread costs of reproduction, particularly in small mammals, as does that associated changes in body mass and organ remodelling required to facilitate this^17^. Oxidative stress has also been suggested as a potentially important cost of reproduction^18^,^19^, which might be linked to increased glucocorticoids^20^ or metabolism^17^, and could be generated by allocation during pregnancy and lactation (but see [18] for a critical discussion), or conceivably from interactions with males.

At two time points over the pairing period (three days and two weeks after pairing), females were sampled for body weight, faecal corticosterone metabolites (FCM), metabolic rate, and respiratory exchange ratio (RER). RER provides an indication of metabolism of carbohydrates or fats for energy metabolism^21^. An RER of close to one indicates almost exclusive oxidation of carbohydrates for energy metabolism, while increasingly lower RERs indicate greater oxidation of fat^22^. After housing partners were removed all females were left for a further four weeks, which corresponded to the end of weaning for females that had given birth to young (e.g. those housed with intact males). At this point females were again sampled for FCM and body weight, and then were culled to measure oxidative stress in liver and kidney, two tissues where changes in markers of oxidative stress have been noted with reproduction in mice^23^.

## Results

### Body weight

**Figure 1:**
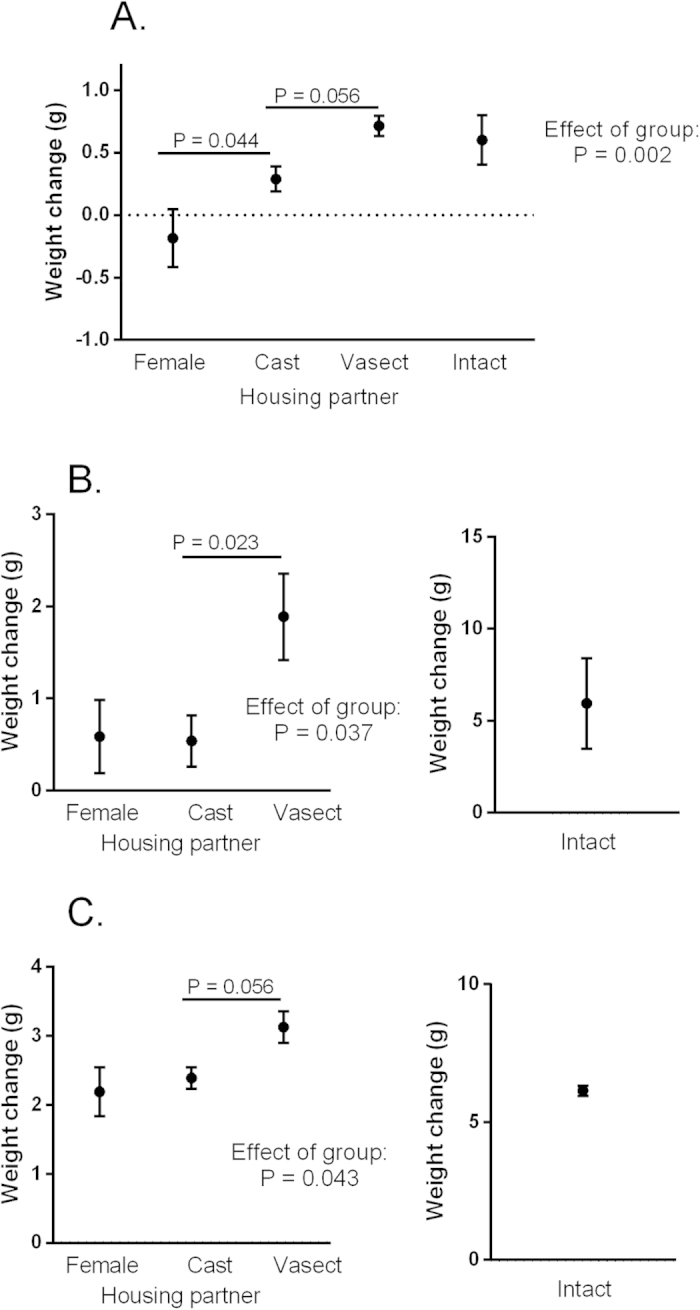
Influence of male gonadal status on female weight gain over the experiment, after three days of pairing (**A**) two weeks of pairing (**B**) and four weeks after males were removed (**C**). Females where housed with either other females (Female), castrated males (Cast), vasectomised males (Vasect), or intact males (Intact). P values indicate results from planned contrasts, run after testing for an overall effect of group using a GLM. Data are displayed as means ± s.e.m.

Female weight changed over the experiment in a manner that was dependent on housing partner (interaction between time and housing partner: F_6,54_ = 3.11, P = 0.011). After three days of pairing, females housed with different partners showed significant differences in weight gain (F_3,30_ = 6.64, P = 0.002; Fig. 1A). Planned comparisons revealed that females housed with other females were lighter than those housed with castrated males. Fourteen days after pairing most of the females with intact males were pregnant, so weight change in this group is clouded by their carrying of offspring, and these females were excluded from further analysis (although data is shown for reference in Fig. 1). Non-pregnant females housed with different partners still showed significant differences in weight at this time point, with females housed with vasectomised males being significantly heavier than those housed with castrated males (F_2,23_ = 3.88, P = 0.037; Fig. 1B). An overall difference in weight between females of the three non-pregnant groups (e.g. housed with other females, castrated and vasectomised males) was still observable at the end of the study (F_2,23_ = 3.67, P = 0.043; Fig. 1C), four weeks after males were removed. Planned contrasts failed to fully resolve the nature of this difference, although these comparisons tentatively suggest that differences in weight gain between females housed with vasectomised and castrated males might extend to this period, although the difference was not significant (P = 0.056).

### Faecal Corticosterone Metabolites

**Figure 2:**
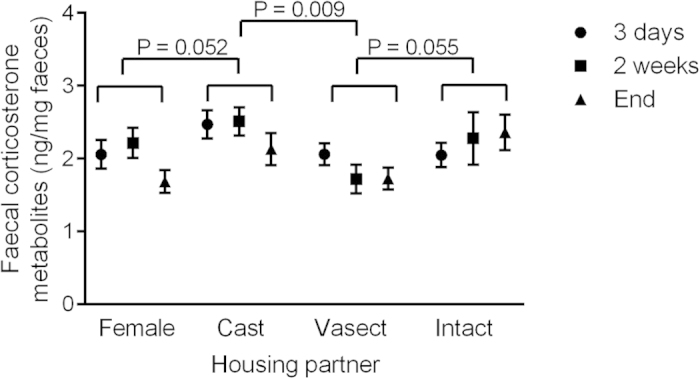
Concentrations of faecal corticosterone metabolites in females housed with females or males of different gonadal status. Females where housed with either other females (Female), castrated males (Cast), vasectomised males (Vasect), or intact males (Intact). P values indicate results from planned contrasts, run with a repeated measures GLM including values for each individual from samples collected three days or two weeks after pairing, or at the end of the experiment. Data are displayed as means ± s.e.m.

FCM levels did not change over time (effect of time: F_2,54_ = 1.40, P = 0.26; interaction between time and housing partner: F_6,54_ = 1.19, P = 0.33), but females housed with different types of con-specifics showed consistently different FCM levels over the study (effect of housing partner: F_3,27_ = 3.15, P = 0.040; Fig. 2). Particularly notable were the significantly lower FCM levels in females housed with vasectomised males compared to females with castrated males (P = 0.009). In addition to this significant between group contrast, the two other between group comparisons approached significance and are worth noting. Females housed with intact males tended to (P = 0.055) have higher FCM levels than those females housed with vasectomised males (Fig. 2). This trend fits with previous observations that pregnancy and lactation increase corticosterone levels. Additionally, females housed with castrated males showed a trend (P = 0.052) for higher FCM than females housed with other females, although again, this effect did not reach conventional statistical significance (Fig. 2).

### Metabolic rate and respiratory exchange ratio

**Figure 3:**
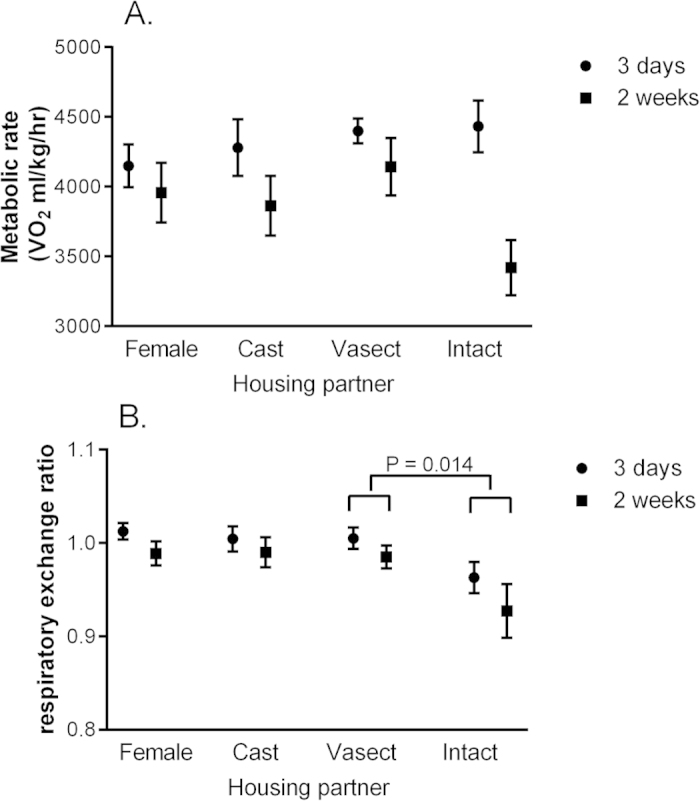
Metabolic rate and respiratory exchange ratio in females housed with females or males of different gonadal status. Females where housed with either other females (Female), castrated males (Cast), vasectomised males (Vasect), or intact males (Intact). P values indicate results from a planned contrast, run with a repeated measures GLM including values for each individual from samples collected three days or two weeks after pairing. Data are displayed as means ± s.e.m.

Metabolic rate was influenced by body weight (effect of body weight as a covariate: F_1,53_ = 4.36, P = 0.042), but did not differ significantly between groups at three days or two weeks after pairing (Effect of group with body mass included as a covariate: F_3,53_ = 0.58, P = 0.63; Fig. 3A). As reproduction also has an impact on the utilisation of substrates for energy metabolism, we further calculated RER. Females paired with other females, or castrated or vasectomised males, had a RER of close to one (Fig. 3b), indicating an almost exclusive reliance on carbohydrates for energy metabolism. RER was lower in females paired with intact males - those females that where pregnant over the study - than vasectomised males, highlighting that these females showed an increased usage of fat for energy metabolism (effect of housing partner: F_3,26_ = 3.52, P = 0.029; Fig. 3B).

### Oxidative stress

Mice were culled four weeks after male removal, corresponding to the end of lactation for reproducing females, and several markers of oxidative stress assessed in liver and kidney. No consistent changes in oxidative damage markers were observed in relation to a female’s housing partner (Table 1). Oxidised glutathione in kidney differed between groups, with levels tending to be lower in females housed with castrated males. However, the lack of concordance with other markers of oxidative stress highlights that oxidative stress does not consistently change with reproduction, at least in these tissues, and is not consistently influenced by housing partner.

## Discussion

Housing females with males of different gonadal status influenced patterns of weight gain and altered faecal corticosterone metabolites, but did not detectably alter patterns of oxidative stress in liver and kidney. Investment in offspring, which occurred only when females where housed with intact males, induced physiological and metabolic changes associated with carrying and allocation to offspring, including increased body mass, altered substrate metabolism, and a tendency for elevated FCM. These effects of pregnancy are consistent with those reported previously^13^,^14^,^17^,^24^. Our experimental design also allowed us to detect several effects of being housed with a male that are independent of the effects of offspring production, highlighting that a male’s presence can influence aspects of female physiology that may be linked to the rewards and/or costs associated with mating and male presence.

Females housed with a male (instead of a female) showed a greater increase in body weight over the experiment. This was detected at one measured point for all the male-paired groups, although the nature of these between group differences varied over time. For those females housed with castrated males, a significant weight change compared to those housed with females was detected three days after pairing but not thereafter – by two weeks of pairing those housed with females had gained a similar amount of weight to those housed with castrated males. By contrast, those paired with vasectomised males had gained significantly more weight after two weeks than those with castrated males, and showed a similar albeit non-significant trend for increased body weight at the end of the study. Part of this response induced by vasectomised males, early in the pairing, may be a consequence of the induction of pseudopregnancy, since females mated with vasectomised males can become pseudopregnant and show a temporary increase in body mass of approximately 5–10% of their normal body weight^25^,^26^. It’s notable, however, that body weight changes with pseudopregnancy are usually transient, decreasing to original levels by about 20 days after mating^26^. Here, body mass was not observed to decline, and there was still a trend for a difference in weight between those housed with castrated or vasectomised males. Housing with a vasectomised male might, therefore, have some longer-term effects on body weight dynamics that were not observed in previous studies where females were only housed with vasectomised males until they mated (e.g. for 24–48 hours), after which the male was removed. Our experimental design was set up explicitly to interpret differences between the castrated and vasectomized male treatments as due to the consequences of mating and insemination, and so we are led to interpret the observed weight gain in females paired with vasectomized males in this way. It may be that mating-induced injury, or some component of seminal chemistry or microbiology may have induced the response we document here. Further detailed experimental study is justified in order to resolve the causes of the observed effect. It is possible that the effects of these weight changes could extend to influence other aspects of physiology, and other life history traits like future offspring production, although this will need to be the subject of future experimental work.

Females housed with different partners also differed in their corticosterone levels over the study. The trend for higher corticosterone levels in pregnant females (i.e. females paired with intact males), compared to those housed with vasectomised males, is consistent with previous research^13^,^14^, implicating elevated corticosterone in mobilising gestational resources to transfer to offspring. More surprising, females housed with castrated males also showed higher FCM levels than those with vasectomised males, suggesting activation of the hypothalamic-pituitary-adrenal axis (i.e. HPA-axis). Perceiving the presence of a male, but not mating or being solicited to mate, therefore, appears to generate a stress response. This result might reflect a mismatch between sexual anticipation and reward, with the introduction of a male generating an anticipatory increase in corticosterone, but the lack of mating meaning these levels remain consistently high. This draws some interesting parallels with a recent study in *Drosophila* where sexual perception and reward had differential effects on lifespan in males. Detecting the presence of a mate without mating (generated by genetic manipulation of sex-specific pheromones in same sex individuals) is more costly in terms of lifespan than allowing flies to mate in the same context^1^. It must be noted, however, that castrated males and their odors do not induce in females many of the physiological responses that androgenized males do. Castrated males do not influence sexual maturity in juveniles^27^, and cannot induce pregnancy termination in recently impregnated females^28^. Therefore, the female response to castrated males would be driven by some male trait that is not dependent on testosterone. Another possibility to consider for this result, at this stage, is that castrated males represent an “unusual” housing partner, neither a normal female nor male, and this unfamiliar housing partner induces the stress response we observed.

Understanding of the life history and physiological consequences of the alterations in body mass and differential stress responses that we report here requires longer-term monitoring of females and further analysis of the downstream changes in physiology. Our results suggest that, apart from the effects of pregnancy itself, neither metabolic rate nor oxidative stress differ strongly between females paired with males of different gonadal status, even though both of these aspects of physiology are sometimes linked to growth, reproduction and/or elevated glucocorticoids^20^,^29^. The lack of an effect of reproduction on oxidative stress markers was not even observable in the intact group, but this is consistent with previous work, particularly in laboratory conditions. Some authors have actually found oxidative stress to decrease during lactation in small rodents^30^,^31^,^32^, although these effects can be smaller when measuring oxidative stress post-weaning, as conducted here^31^,^32^. We also note that our experiment was designed so as to detect effects of different housing partners that are fairly large, and might have detectable impacts on female survival and future reproduction when followed across longer periods of life. As a consequence, more subtle changes in oxidative stress and metabolism in females with different housing partners may be detectable in future studies with greater power. Future studies that provide additional replication of the body weight and corticosterone effects we observe will also be of benefit. Our between group analytical approach in this study was to define logical planned contrasts prior to experimentation that would allow us to disentangle the effects of mating from those associated with caring for offspring. As advised by some^33^ we then took the approach of not adjusting for type-1 error (e.g. multiple comparisons), allowing us to achieve greater power in an experiment with relatively small sample sizes. Since several of our results were close to the conventional threshold for statistical significance, and would have probably been above this threshold if we had taken a more constraining approach of adjusting for type-1 error, replication of these results will help to confirm the patterns we reveal.

In future studies, it might also be of interest to assess changes in plasma glucose, insulin, and metabolites associated with energy metabolism, since these vary more consistently with both reproduction and elevated glucocorticoids, and might provide a simpler test of whether metabolic effects occur with male presence. We also note that metabolic rate was measured over the first two weeks of the experiment–without housing partners present - and oxidative stress was assessed four weeks after male removal. Differences in oxidative damage may have been observable if we had examined females while they were still paired with males, when stronger differences in weight where observable, although this would have meant euthanizing animals earlier in the study. Similarly, utilisation of a different technique to assess energy expenditure, such as the doubly labelled water method^34^, would allow assessment of female energy expenditure while females are in the presence of and interacting with the males. With the present technique we can only assess female metabolism when females are removed from the cage they share with a male and are placed in the metabolic rate chamber.

In conclusion, these results highlight that simply the presence of a male, independent of mating, can have morphological and endocrine effects on females that may contribute to the costs of reproduction in mice. Our approach of surgically manipulating male interest in mating (castration) and ability to impregnate (vasectomy) may yet uncover longer-term demographic and physiological costs of male mating behaviour and sexual conflict in mammals. To further disentangle the various components of male mating behaviour and ejaculate components that are still present in vasectomised male mice, further surgical manipulations may provide an attractive approach. Surgical removal of the seminal vesicles, for example, would help resolve the contribution of male seminal fluid on female physiology. Understanding whether the act of mating reduces female stress, in comparison to detecting an androgenised male’s presence, might be improved by keeping females in olfactory and visual contact with a male, but inhibiting direct contact with a barrier. Using these techniques, we may further understand how the complexity of male interactions influence costs of female reproduction, over and above the optimal allocation that females desire to make to offspring production.

## Methods

### Animals

This research was approved by the University of New South Wales (UNSW) Animal Care and Ethics Committee, approval 12/129B. The methods were carried out in accordance with these approved guidelines. Mice were of the C57BL/6 strain and were purchased for the Australian BioResource Center (ABR, Mossvale, NSW, Australia). Before being shipped to UNSW, male mice were either castrated, vasectomised or sham vasectomised (intact), when aged between 6–8 weeks old. Animals were then sent to UNSW and allowed three weeks to habituate. Mice were kept in specific pathogen-free conditions, at 22 ± 2 °C on a 12:12-h light/dark cycle, with the dark period starting at 9 am. The light cycle was reversed so that experimental manipulations occurred in the dark period under dim red light.

### Experimental Procedures

After the three week habituation period, experimental females (aged 10–12 weeks old) were each housed with either an unfamiliar female, a castrated male, vasectomised male or an intact male (n = 8 per treatment group). All pairs were housed in open top cages (48 × 11.5 × 12 cm) in the same room, with the cages of females in different groups randomly assorted across two racks. Females in different treatment groups were therefore exposed to equivalent levels of background auditory and olfactory cues emanating from outside the cage they were housed. A tumour was visibly observable in one female housed with an intact male part way through the experiment, so this animal was removed from the study. All females paired with intact males produced offspring that survived until they were weaned at 28 days old. Since we wanted to avoid any infanticide that male mice might cause to newborn young in the intact group, we removed female housing partners when those females housed with intact males were heavily pregnant and we anticipated them giving birth over the next one – two days. When an intact male was removed we removed one of the other housing partners from each of the other treatment groups – these individuals were randomly selected from within each of the other treatment groups. The first housing partners were removed after 19 days of pairing, while the last were removed 32 days after pairing.

Females were sampled at three different points after pairing: three days after being housed with their housing partner, two weeks after pairing, or four weeks after housing partners were removed. At each point, females were removed from their partners at 10 am, weighed, then placed individually in clear plastic cages used for assessment of energy metabolism. These cages contained a mesh grid that the mice stand on, but which allowed faeces to fall to the area below. After two hours, mice were briefly removed from the cages so that faeces (uncontaminated from urine) could be collected for measurement of corticosterone metabolites. After measurement of energy metabolism (see below) animals were returned to their home cages. After the final sampling point, four weeks after partner removal, females were left a further three days, then culled via cervical dislocation and organs quickly removed for measurement of oxidative stress.

### Whole-Body Metabolism

Whole body metabolism was assessed using our published approach with this mouse strain^35^. Indirect calorimetry was measured using the metabolic cage system from Columbus Instruments. Measurements began at 10:00 AM and continued for 12 h. The first 7 h of the measurement period was an acclimatization period and the last 5 h were used for analysis. The calorimeter was calibrated before the experiment with a standard span gas (0.504% CO2, 20.43% O2 balanced with N2) and cross-calibrated with room air. Data were collected every 13 min over this 12-h period. The metabolic measurements included the volume of carbon dioxide produced (VCO_2_), the volume of oxygen consumed (VO_2_), and RER (VCO_2_/VO_2_). The data are represented as the mean values over each 5-h period.

### Faecal Corticosterone Metabolites (FCM)

Feacal samples for FCM analysis were frozen at −80 °C after collection. After defrosting, all samples were dried thoroughly at 60 °C, then homogenised. One ml of 80% aqueous methanol was then added to 0.05 g of powdered faeces and the mixture vigorously shaken for 30 minutes on a multi-vortex. After shaking, the faeces were separated from the methanol by centrifugation for 10 minutes at 2500 g. The methanol supernatant, containing the steroid metabolites, was then transferred into a new vial and the liquid evaporated completely^36^. The dried samples were then sent to the University of Veterinary Medicine in Vienna for analysis. After redissolving in 80% methanol, FCM were analysed in an aliquot of the extract with a 5α-pregnane-3ß,11ß,21-triol-20-one enzyme immunoassay, previously developed and validated for mice (details see^37^,^38^).

### Oxidative stress

Oxidative stress was assessed using our previously published methods^31^,^32^,^39^. Protein thiols are groups on proteins that are susceptible to oxidation, with a decrease in concentration indicating protein oxidation^40^. Protein thiols were measured as described by Di Monte *et al*.^41^ altered for use on a plate reader^42^. Aconitase is an enzyme that is very susceptible to deactivation by the superoxide radical and can be used as a marker of uncontrolled ROS production. The activity levels of this enzyme were assessed according to^31^. Glutathione is an important intracellular thiol antioxidant. This antioxidant is readily oxidised under periods of oxidative stress and the ratio of total to oxidised glutathione can be used as a marker of oxidative stress^43^,^44^. Total and oxidised glutathione were measured using the automated glutathione recycling assay^44^ modified for use on a plate reader^42^.

### Data analysis

Statistical tests were carried out with SPSS version 22. Repeated measures GLMs assessed the changes in weight, FCM and metabolism over time, whether there were differences between females housed with different partners, and whether there was an interaction between these two factors. Univariate GLMs tested for differences between females with different partners for the different measures of oxidative stress, and for differences in weight at individual time points. We used the repeated contrast function in SPSS to test our planned contrasts between specific groups when univariate GLMs proved significant. The three planned contrasts were: 1. Between those females housed with females and those females with castrated males. 2. Between those with castrated males and those with vasectomised males. 3. Between those with vasectomised males and those with intact males.

## Additional Information

**How to cite this article**: Garratt, M. *et al*. Male Presence can Increase Body Mass and Induce a Stress-Response in Female Mice Independent of Costs of Offspring Production. *Sci. Rep.* **6**, 23538; doi: 10.1038/srep23538 (2016).

## Figures and Tables

**Table 1:** Markers of oxidative stress in females after being housed with females or males of different gonadal status.

Marker	Mean ± SE				Effect of group		
	Control	Cast	Vasect	Intact	df	F	P
Liver Thiols	97.2 ± 8.1	90.1 ± 6.4	129.5 ± 15.3	108.8 ± 23.7	3,29	1.72	0.19
Kidney Thiols	65.0 ± 4.0	63.4 ± 1.9	63.3 ± 3.1	66.8 ± 2.4	3,30	0.75	0.53
Liver Aconitase	0.19 ± 0.02	0.17 ± 0.04	0.18 ± 0.04	0.22 ± 0.06	3,30	0.35	0.79
Kidney Aconitase	0.13 ± 0.009	0.15 ± 0.014	0.14 ± 0.010	0.14 ± 0.008	3,28	0.4	0.75
Liver GSH	40.2 ± 4.4	35.4 ± 4.3	46.2 ± 7.9	49.5 ± 14.5	3,30	0.55	0.65
Kidney GSH	7.59 ± 3.11	11.91 ± 2.93	7.56 ± 1.8	11.09 ± 3.88	3,30	0.6	0.62
Liver GSH/GSSH	0.0322 ± 0.004	0.0325 ± 0.007	0.0391 ± 0.007	0.0326 ± 0.008	3,30	0.29	0.84
Kidney GSH/GSSH	0.0611 ± 0.010	0.0327 ± 0.002	0.0534 ± 0.007	0.0452 ± 0.004	3,30	3.59	0.027

GSH represents glutathione, GSSH the proportion of glutathione in the oxidised form. Only the proportion of oxidised glutathione in the kidney showed any evidence of differing between groups, with females that were housed with castrated males having lower levels of oxidation those housed with females (Planned contrast: P = 0.004) and vasectomised males (P = 0.030).
